# Modeling *Candida auris* skin colonization: Mice, swine, and humans

**DOI:** 10.1371/journal.ppat.1010730

**Published:** 2022-09-08

**Authors:** Emily F. Eix, Jeniel E. Nett

**Affiliations:** Departments of Medicine and Medical Microbiology & Immunology, University of Wisconsin-Madison, Madison, Wisconsin, United States of America; Geisel School of Medicine at Dartmouth, UNITED STATES

## What is *Candida auris* skin colonization?

The recently emergent fungal pathogen *Candida auris* frequently persists on skin of patients and can cause invasive disease with mortality rates over 50% [1,2]. It is the first fungal pathogen to be labeled as an urgent global public health threat due to its high capacity for person-to-person spread and its ability to produce recalcitrant, drug-resistant infection [1–5]. Since identification in 2009, *C*. *auris* has rapidly spread around the world, now accounting for 20% to 30% of *Candida* bloodstream infections in some healthcare settings [6,7]. Prior to development of invasive disease, *C*. *auris* colonizes patients, proliferating on the skin and at other nonsterile sites [4,6]. Of patients colonized with *C*. *auris*, approximately 95% involve the skin [8]. In a study of critically ill patients admitted to an intensive care unit, *C*. *auris* bloodstream infection developed in approximately 25% of patients within 60 days following skin colonization [8]. This pathogenicity pattern distinguishes *C*. *auris* from other *Candida* species, including *Candida albicans*, which typically reside as commensals in the gastrointestinal tract prior to the development of disseminated disease.

*C*. *auris* frequently colonizes the axilla and groin, sites typically sampled in the screening of patients for resistant bacteria, including methicillin-resistant *Staphylococcus aureus* [9–11]. However, a broader investigation of residents in a skilled nursing facility identified *C*. *auris* colonization across 10 different body sites, with frequent colonization of 2 or more areas [12]. While the investigators similarly detected *C*. *auris* from axilla and groin samples, other colonization sites included the nares, fingertips, palms, toe webs, and perianal skin. *C*. *auris* appears to persist at a variety of patient skin sites for many months. With a breach of the skin barrier, *C*. *auris* can enter the bloodstream and produce invasive disease. Such breaches commonly occur in hospitalized patients who routinely undergo vascular catheter placement, gastrostomy tube insertion, and/or other surgical procedures, all of which correlate with invasive *C*. *auris* infection [4,13]. In addition, the skin of colonized patients (intact or desquamated) regularly contacts shared medical equipment and other surfaces, which appear to contribute to continued nosocomial transmission [2,4,14].

## What models currently exist to study *C*. *auris* skin colonization?

**Fig 1:**
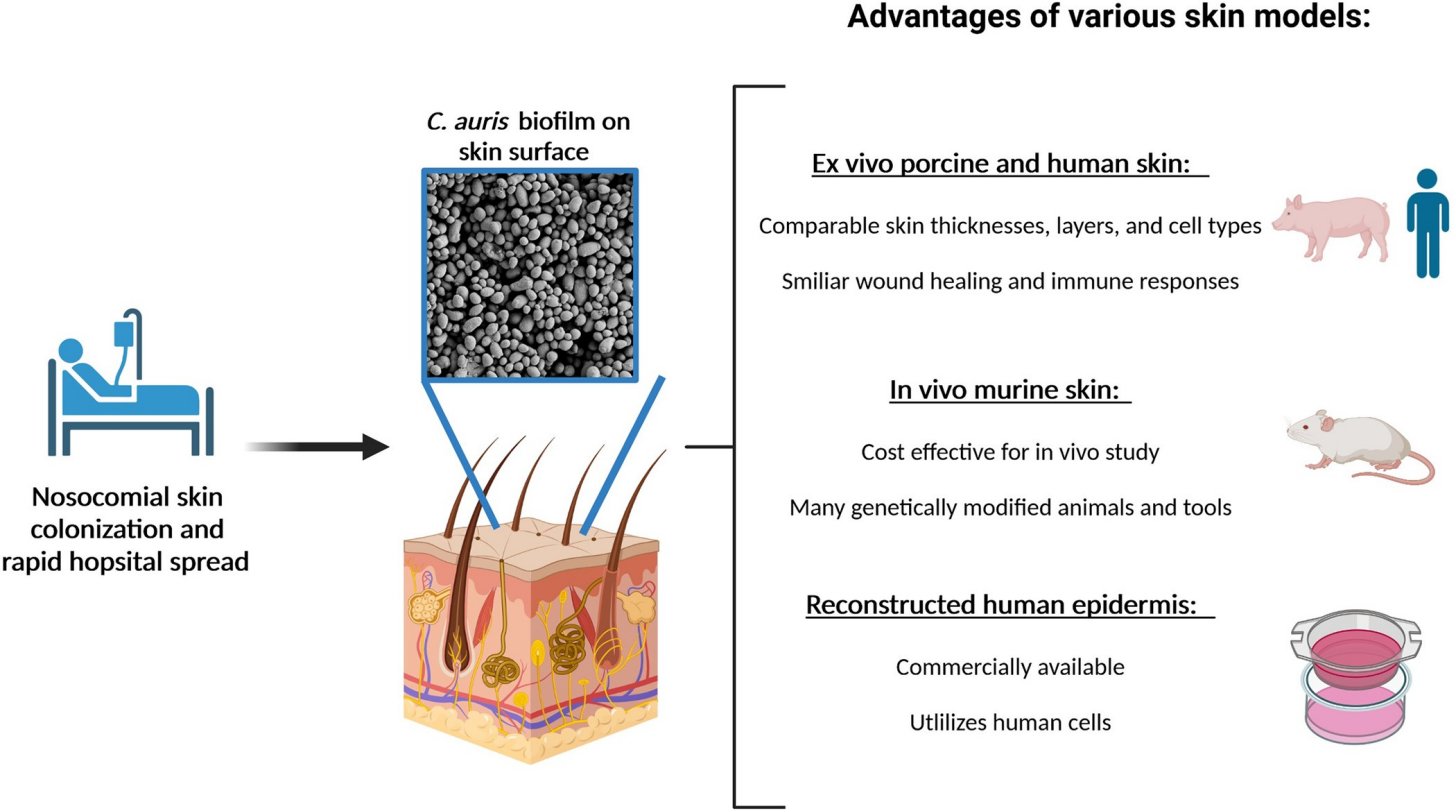
Modeling *C*. *auris* skin colonization. *C*. *auris* spreads rapidly in healthcare settings and proliferates on patient skin, leading to severe disease. Skin colonization can be modeled using ex vivo human and porcine skin, in vivo using mice, and with reconstructed human epidermis. Each of these models has its own advantages and limitations. *C*. *auris* biofilm growth on the surface of ex vivo human skin was imaged by scanning electron microscopy. Created with BioRender.com.

Murine models, widely employed for study of fungal pathogenesis, have also been developed for examination of *C*. *auris* on skin, largely examining ear pinnae and/or shaved back sites (Fig 1) [15,16]. Huang and colleagues show that *C*. *auris* readily colonizes the ear pinnae of immunocompetent C57Bl/6NTac mice without causing signs of inflammation or infection [15]. Imaging and microbiologic analysis reveal a reservoir of *C*. *auris* residing in deeper skin layers and within hair follicles [15]. The observation may help explain how patients can remain persistently colonized with *C*. *auris* despite negative testing of the skin surface. Consistent with clinical observations of colonization, other clades of *C*. *auris* also readily colonize murine pinnae, in contrast to *C*. *albicans*, which is much less efficient in colonization. In this model, control of *C*. *auris* colonization appears dependent on IL-17 receptor signaling that is triggered by several lymphoid cell populations, including T-cells and innate lymphoid cells. T-cell and ILC-deficient mice exhibited greater *C*. *auris* skin colonization that persisted for longer than wild-type mice, pointing to the importance of these immune cells in controlling *C*. *auris* colonization. In a similarly designed study, Ghannoum and colleagues found *C*. *auris* to colonize the ear pinnae and shaved backs of BALB/c mice without inducing signs of inflammation or evidence of invasive disease [16]. They developed the model to analyze treatment efficacy for topical antifungals, as was shown for proprietary formulations of terbinafine and clotrimazole. The group has also adapted a guinea pig skin model of *C*. *auris* infection for study of antifungal efficacy [17].

Ex vivo models for both human and porcine skin appear to recapitulate the high capacity of *C*. *auris* to colonize patients [18–20]. In these models, skin remains viable in tissue culture media for days and *C*. *auris* replicates on the epidermal surface [18–20]. *C*. *auris* similarly colonizes the surface of both human and porcine skin, forming robust multilayer biofilms [18,20]. *C*. *auris* appears to colonize the outer epidermal layers without invading deeper tissues. A variety of *C*. *auris* isolates, including those from different geographic clades, demonstrate comparable capacities to colonize ex vivo skin and grow to burdens over 10-fold greater than *C*. *albicans*. This does not appear to be due to enhanced thermotolerance of *C*. *auris*, as both species grow similarly at 37°C [21]. Porcine skin exhibits anatomical resemblance to human skin and is frequently employed for skin modeling [22,23]. Humans and swine have comparable skin thicknesses and layers, while rodent skin is much thinner and more loosely attached to subcutaneous tissue [22]. Mice also differ in aspects of immune response and wound repair mechanisms [24]. However, murine models offer an expansive number of genetically modified animals and are more widely available for in vivo study due to size and cost.

In addition to these in vivo and ex vivo models, Brown and colleagues has described a model of reconstructed human epidermis for study of *C*. *auris*-infected wounds. Transcriptional studies revealed changes to host gene expression in a *C*. *auris* colonized wound as well as up-regulated *C*. *auris* virulence genes in this 3D coculture system [25]. This skin model is useful as a commercially available system that utilizes human cells and can be used to uncover fungal phenotypes as well as host responses.

## What have we learned from these models?

Animal models have helped us understand how *C*. *auris* persists on the skin of patients despite routine bathing and decolonization attempts [2,12,26]. Although *C*. *auris* exhibits in vitro susceptibility to one of the most commonly used hospital antiseptics, chlorhexidine, multiple studies demonstrate that patients can remain colonized following daily topical application of this agent [2,4,12,26]. This phenomenon has been analyzed using both ex vivo porcine skin and in vivo mouse models. When used as a topical prophylactic regimen on the skin of mice, chlorhexidine can prevent *C*. *auris* colonization [15]. However, this preventative activity decreases when animals are challenged with a high burden of *C*. *auris* and organisms can colonize the skin. Similarly, chlorhexidine treatment of mice with established colonization reduces the viable burden (2 log reduction) but does not completely sterilize the site [15]. Chlorhexidine treatment of porcine skin with *C*. *auris* colonization also reduces fungal burden, but by a more modest amount (0.5 log reduction) [19]. This is in contrast to in vitro conditions where similar chlorhexidine treatment leads to a 2 log reduction and typically eliminates in vitro regrowth. In both murine and porcine models, *C*. *auris* appears to reside in deeper tissues, such as the hair follicles [15,19]. While chlorhexidine can reduce the burden of *C*. *auris* on skin, it does not appear to eradicate the organism, allowing for fungal regrowth and persistent colonization, mirroring clinical observations. Other antiseptics including isopropanol, tea tree oil, and lemongrass oil can improve the activity of chlorhexidine against *C*. *auris* on porcine skin [19]. However, it is unclear if these combinations would improve decolonization attempts in healthcare settings.

Skin models illustrate the heightened capacity of *C*. *auris* to colonize skin compared to other species in the genus. While other *Candida* spp. can colonize skin, healthcare-associated outbreaks due to these species are rare. In mouse and porcine skin models, *C*. *auris* proliferates to burdens 10- to 100-fold greater than *C*. *albicans*. Phylogenetically *C*. *auris* is closely related to *Candida haemulonii* and *Candida duobushaemulonii*. On both human and porcine skin, *C*. *haemulonii* and *C*. *duobushaemulonii* exhibit significant growth defects compared to *C*. *auris*, despite their close genetic similarity [20]. These findings parallel clinical observations that demonstrate *C*. *auris* has an enhanced capacity for growth on patient skin compared to other *Candida* species and underscore the utility of these models for mechanistic and therapeutic study of *C*. *auris* colonization.

## How can these models be used for future study?

While *C*. *auris* skin colonization is not marked by tissue invasion, wounding represents a mode for organisms to enter deeper tissues and cause invasive disease. Common examples of wounds in the hospital setting include surgical incisions, burn wounds, and vascular catheter insertions. Understanding the interface of *C*. *auris* colonization and wound infection may shed light on strategies to prevent invasive disease. Mechanical wounding can be incorporated into the mouse, guinea pig, human, and porcine skin models of *C*. *auris* colonization models [25,27,28]. To mimic the wounding produced by catheter insertion, animal models of vascular catheter infection, such as a rat model, can be utilized for study of *C*. *auris* [28]. These and other models will be important for further dissection of *C*. *auris* growth in the setting of nosocomial skin wounding.

The importance of the skin microbiome in health and disease has become increasingly recognized over the past several decades. *C*. *auris* outbreaks also highlight the need to understand the skin mycobiome in this context. Huang and colleagues utilized metagenomic sequencing to characterize the skin microbiome of skilled nursing facility patients during a *C*. *auris* outbreak [9]. They found the patients in the outbreak facility were more likely to harbor a variety of *Candida* spp., when compared to individual residing in a non-outbreak facility, whose mycobiomes were more likely to be dominated by *Malassezia*. In addition, the patients colonized with *C*. *auris* exhibited dysbiotic skin microbiomes with abundant nosocomial pathogens, including *Acinetobacter*, *Klebsiella*, and *Enterococcus*. Investigation using skin colonization model systems may shed light on how these organisms or other skin microbiota influence *C*. *auris*, including potential antagonistic or synergistic interactions between organisms.

While skin models have helped elucidate the process of *C*. *auris* colonization, much remains unknown about this recently emerged pathogen. As *C*. *auris* continues to spread and cause disease in healthcare settings worldwide, understanding the mechanisms of skin colonization and pathogenicity remains critical. Continual development and use of skin models are important to further our understanding of *C*. *auris* growth in this niche.
